# The Institutional Worker

**Published:** 1912-02-10

**Authors:** 


### INSTITUTIONAL WORKER

**Being a Special Supplement to "The Hospital"**

### Editor's Notices.

**Contributions:**

Contributions should be written, or preferably typed, on one side of the paper only, and all articles sent in are accepted upon the distinct understanding that they are forwarded to THE HOSPITAL only.

The Editor cannot undertake to return MSS. not used, but when a stamped directed envelope is enclosed the MSS. may be returned if a special request is made.

Accepted articles and paragraphs of news will be paid for after publication at the scale rate.

**Address:**

To prevent delay all contributions and letters on editorial business must be addressed exclusively to the Editor, "The Hospital," 29 Southampton Street, Strand, London, W.C. It is important that this regulation shall be strictly observed.

**Correspondence:**

Correspondence on all subjects is invited. The name and address of correspondents must be given as a guarantee of good faith, but not necessarily for publication.

**Special Articles:**

Special articles are invited, and questions, inquiries, and paragraphs upon all matters relating to the work, administration, and management of general, special, mental, fever, cottage and convalescent hospitals, sanatoria, homes, institutions, societies and organisations for the treatment or care of the sick, injured and dependents of all classes. Every matter affecting the interests and welfare of the staff of all grades working in these institutions will receive special consideration.

**Administrative Medicine, Research and Items of News:**

Special terms will be paid for approved articles on subjects in this wide field by experts who have made some section of it their special study and interest. We wish to give prominence to every movement tending to advance accuracy of diagnosis, efficiency in treatment, and the development of modern methods to eradicate disease and promote the welfare of the suffering.

**A Bureau of Information:**

We invite inquiries and applications for information and help in providing for that numerous class of sufferers who require special care or house-room which they cannot obtain in their own homes or secure for themselves. We cannot, however, prescribe or recommend practitioners.

**Books for Review:**

Publishers are particularly requested to send advance proofs of any new books of importance whenever possible, as the Editor has made arrangements to publish immediate reviews on a new plan.

**Photographs, Plans, Blocks, and Illustrations:**

It is requested that wherever possible MSS. may be accompanied by illustrations in any of the above forms. The name of the sender and of the article to which it belongs should be written on the back of each photograph, drawing, or block for purposes of identification.

**Local Papers:**

Newspapers containing reports or news paragraphs should be marked and addressed to the Sub-Editor.

**The Coupon System:**

A coupon will be found at the bottom of the third inside page of the cover each week. This coupon must be attached to every question or inquiry to which an answer is desired in THE HOSPITAL.

### Manager's Notices.

Letters relating to the publication, sale, and advertising departments of THE HOSPITAL must be addressed to the Manager, "The Hospital," The Hospital Building, 28 and 29 Southampton Street, Strand, London, W.C.

**Subscriptions** which may begin at any time, are payable in advance. Cheques and Post-Office orders, crossed London County and Westminster Bank, Covent Garden branch, should be made payable to the Manager of THE HOSPITAL and addressed to him at The Hospital Building, 28 and 29 Southampton Street, Strand, London, W.C.

**Advertisements:**

All advertisements for THE HOSPITAL must reach the Manager not later than Wednesday morning in each week. For scale of charges see pages v and 2.

**Institution Authorities.**

THE economical management of an Institution depends very greatly upon purchasing supplies in the cheapest market.

IT is often impossible locally to secure the highest quality at the lowest prices.

IN consequence, a section will be reserved in this part of the paper for announcements of Institutions inviting Tenders for Contracts.

THIS will enable Authorities of Institutions to secure tenders for supplies from all over the country, and ensure purchases at the lowest possible prices consistent with quality.

THE charge for Tenders is 6s. per inch, and for this small outlay many pounds may be saved on supplies of all descriptions.

**Special Rates** will be quoted for those institutions which agree to send all their vacancy, contract, and public notices to THE HOSPITAL each year, so as to make it a file of such announcements for ready reference.

**Classified Advertisements:**

All small advertisements will be classified, for this section of the paper is widely read and of special interest. We wish to encourage those wanting appointments who are attracted to institutional work, and therefore offer them the reduced rate of *twenty-one words and under for* 1s., and *for every ten and part of ten words beyond*, 6d.

This form may be used for small prepaid advertisements, and when filled up should be posted to the

*Manager*, THE HOSPITAL,
28 and 29 Southampton Street,
Strand, W.C.

Appointments Wanted, 21 words ... 1s. 0d.
Appointments Vacant, 21 words ... 2s. 6d.

| | | |
|---|---|---|
| | | |
| | | |
| | | |
| | | |
| | | |
| | | |
| | | |

### OFFICIAL APPOINTMENTS VACANT.

### Poor-Law Vacancies.

Assistant Cook (£22), Islington Guardians. Matron of the Infirmary, Highgate Hill, N.
Assistant Cook (£20), Bethnal Green Guardians. Matron of the Workhouse, Waterloo Road, Victoria Park, N.E.
Assistant Female Attendant (£25), Bristol Guardians. Mr. J. J. Simpson, C. to G., Bristol. Feb. 10.
Assistant Female Attendant (£16), St. Pancras Guardians. Matron of the Workhouse, King's Road, N.W.
Assistant Labour Master (£35), Brentford Union. Mr. W. Stephens, C. to G., Isleworth, W. Feb. 13.
Assistant Laundress (£20), Holborn Union. Master of the Workhouse, Mitcham, Surrey. Feb. 12.
Assistant Laundress (£23), Kensington Guardians. Matron of the Workhouse, Marloes Road, Kensington.
Assistant Master (£50), Kensington Guardians. Master of the Workhouse, Marloes Road, Kensington, W. Feb. 14.
Assistant Matron (£25), Uppingham Union. Mr. F. Oakley, C. to G., Uppingham. Feb. 12.
Assistant Nurse (£26), Lincoln Union. Mr. W. B. Danby, C. to G., Lincoln. Feb. 12.
Assistant Nurse (£22 10s.), Watford Union. Mr. F. Wilson, C. to G., Watford. Feb. 13.
Assistant Nurse (£30), Cuckfield Union. Mr. E. J. Waugh, C. to G., Hayward's Heath. Feb. 15.
Assistant Nurse (£25), Bedford Union. Mr. W. Payne, C. to G., Bedford. Feb. 19.
Assistant Nurse (£22), Parish of Bermondsey. E. Pitts Fenton, C to G, 283 Tooley Street, S.E. Feb. 19.
Assistant Nurse (£20), Godstone Union. E. Alston Head, C. to G., East Grinstead. Feb. 15.
Assistant Nursery Attendant (£17), Holborn Union. Matron of the Workhouse, 1a Shepherdess Walk, N.
Assistant Overseer (Valuer) (£500), Birmingham T.C. The City Treasurer, Birmingham. Feb. 10.
Assistant Relieving Officer (£100), Marylebone Guardians. Mr. H. T. Dudman, C. to G., Northumberland Street, Marylebone Road, W. Feb. 17.
Barber and Bath Attendant (35s. weekly), Poplar and Stepney Sick Asylum. Mr. W. R. Foskett, Clerk to the Managers, Divans Road. Bromley-by-Bow, E. Feb. 15.
Barber and Bathman (£25), Hastings Union. Mr. A. R. Inskipp, C. to G., Hastings. Feb. 14.
Barber and Bathman (£30), Rotherham Union. Mr. W. C. Harrison, C. to G., Rotherham. Feb. 17.
Charge Nurse (£32), Walsall Union. A. H. Lewis, C. to G., 29 Leicester Street, Walsall. Feb. 20.
Charge Nurse (£32), Rochdale Union. Mr. R. A. Leach, C. to G., Rochdale. Feb. 17.
Charge Nurse (£30), Oldham Union. H. A. Quarmby, C. to G., Rochdale Road, Oldham. Feb. 13.
Charge Nurse (£30), Staines Union. Mr. F. Hutchinson, C. to G., Ashford, Middlesex. Feb. 12.
Charge Nurses (£30), Birkenhead Union. Mr. J. Carter, C. to G., Birkenhead. Feb. 13.
Charge Nurse (£28), Holborn Union. Matron of the Workhouse, 1a Shepherdess Walk, N.
Charge Nurse (£30), Norwich Guardians. Mr. E. J. W. Huggins, C. to G., Norwich.
Charge Nurse (£35), Bromley Union, Kent. E. Haslehurst, C. to G., Union Offices, Park House, Bromley, Kent. Feb. 19.
Charge Nurses (two) (£31 to £35, according to qualifications), East Preston Union. Mr. A. Shelley, C. to G., Littlehampton. Feb. 26.
Clerk and Boys' Attendant (£27 10s.), Nottingham Guardians. Mr. G .M. Howard, C. to G., Nottingham. Feb. 13.
Female Assistant Relieving Officer (£100), Paddington Guardians. Mr. H. F. Aveling, C. to G., 313-319 Harrow Road, W. Feb. 12.
Female Assistant Relieving Officer and Infant Protection Visitor (£50), Sevenoaks Union. F. H. Vibert, C. to G., Bank Chambers, Sevenoaks. Feb. 14.
Female Attendant (£24), Cardiff Union. Mr. A. J. Harris, C. to G., Cardiff. Feb. 12.
Female Attendant and Sempstress (£22), Tamworth Union. Mr. J. H. Dewes, C. to G., Tamworth. Feb. 12.
Female Attendant (temporary) (50s. monthly), Richmond Union. Master of the Workhouse, Richmond, Surrey.
Female Attendant (£22), Bermondsey Guardians. Mr. E. Pitts Fenton, C. to G., 283 Tooley Street, S.E. Feb. 12.
Female Attendants (£25), West Ham Union. Mr. T. Smith, C. to G., Leytonstone, N.E. Feb. 14.
Female Cook (£30), Bermondsey Guardians. Mr. E. Pitts Fenton, C. to G., 283 Tooley Street, S.E. Feb. 12.
Female Mental Ward Attendant (£25), Edmonton Union. F. Shelton, C. to G., Lower Tottenham. Feb. 14.
Foster-Mother (£22), Cardiff Union. Mr. A. J. Harris, C. to G., Cardiff. Feb. 23.
Foster-Mothers (£25), Nottingham Guardians. Mr. G. M. Howard, C. to G., Nottingham. Feb. 13.
Foster-Parents (married couple), (£25 and £20), Bedford Union. Mr. W. Payne, C. to G., Bedford. Feb. 15.
Girls' Caretaker (£30), Basford Union. Mr. H. Stone, C. to G., Basford, Notts. Feb. 12.
Head Attendant on Imbecile Men (£35), Bristol Guardians. Mr. J. J. Simpson, C. to G., Bristol. Feb. 10.
Head Nurse (£29), Bridport Union. J. J. Roper, C. to G., 74 East Street, Bridport. Feb. 13.
Infants' Nurse (£30), Bridgend and Cowbridge Union. R. Harmar Cox, C. to G., Union Offices, Bridgend, Glam. Feb. 24.
Male Attendant (£40), Kingston Union. Mr. C. W. Dash, C. to G., Kingston-on-Thames. Feb. 12.
Male Attendant (£35), Shoreditch Guardians. Mr. R. Clay, C. to G., 213 Kingsland Road, N.E. Feb. 10.
Male Attendant and Barber (£25), Tamworth Union. Mr. J. H. Dewes, C. to G., Tamworth. Feb. 12.
Master's Clerk (£24), Romford Union. Mr. C. Bloomfield, C. to G., Romford. Feb. 12.
Master's Junior Clerk (£15), Wrexham Union. Mr. J. B. Bury, C. to G., Wrexham. Feb. 13.
Master and Matron (£150 and £70), Wandsworth Union. Mr. F. W. Piper, C. to G., St. John's Hill, Wandsworth, S.W. Feb. 17.
Master and Matron (£85 and £55), Milton Union. Mr. E. C. Harris, C. to G., Sittingbourne. Feb. 19.
Maternity Sister (£35), Oldham Union. H. A. Quarmby, C. to G., Rochdale Road, Oldham. February 13.
Matron's Assistant (£20), Bakewell Union. Mr. A. Hawes, C. to G., Bakewell. Feb. 10.
Matron of Children's Home (£30), Bedford Union. Mr. W. Payne, C. to G., Bedford. Feb. 15.
Night Sister (£35), Stockport Union. Matron of the Workhouse, Shaw Heath, Stockport.
Nurse (£30), Aylsham Union. Mr. H. J. Gidney, C. to G., Aylsham, Norfolk. Feb. 12.
Nurse (£27 10s.), Stockbridge Union. Matron of the Workhouse, Stockbridge.
Nurse (£30), Isle of Thanet Union. C. Taylor, C. to G., Minster, nr. Ramsgate. Feb. 22.
Nurse (£35), Festiniog Union. Mr. T. Roberts, C. to G., Portmadoc. Feb. 19.
Nurse (£30), Godstone Union. E. Alston Head, C. to G., East Grinstead. Feb. 15.
Nurses (£30), Wellington (Salop) Union. R. Gwynne, C. to G., Edgbaston House, Wellington, Salop. Feb. 15.
Nurses (two) (£32 each), Leigh Union. Mr. A. H. Hayward, C. to G., Leigh. Feb. 14.
Nurses (£32), Leigh Union. A. H. Hayward, C. to G., Union Offices, Leigh. Feb. 14.
Nurses (£20), St. Pancras Guardians. J. E. P. Hall, C. to G, Town Hall, Pancras Road, N.W.
Porter and Portress (married couple) (£35 joint), East Retford Union. Mr. J. G. Dimock, C. to G., East Retford.
Probationer (£13), West Ham Union. Mr. T. Smith, C. to G., Leytonstone, N.E.
Probationer Nurse (£10), Cheadle Union. F. S. Cox, C. to G., Cheadle, Stoke-on-Trent. Feb. 14.
Probationers (£10), Steyning Union. Mr. A. Flowers, C. to G., Shoreham-by-Sea.
Probationers (£8), Leicester Guardians. Matron of the Infirmary, North Evington, Leicester.

